# Evaluation of Factors Affecting Antimicrobial Activity of Bacteriocin from *Lactobacillus plantarum* Microencapsulated in Alginate-Gelatin Capsules and Its Application on Pork Meat as a Bio-Preservative

**DOI:** 10.3390/ijerph16061017

**Published:** 2019-03-20

**Authors:** Ngoc Thuy Trang Le, Long Giang Bach, Duy Chinh Nguyen, Tran Hong Xuan Le, Khanh Hung Pham, Dai Hai Nguyen, Thai Thanh Hoang Thi

**Affiliations:** 1 Graduate University of Science and Technology, Vietnam Academy of Science and Technology, Hanoi 100000, Vietnam; thuytranglengoc@gmail.com (N.T.T.L.); nguyendaihai@iams.vast.vn (D.H.N.); 2 Institute of Applied Materials Science, Vietnam Academy of Science and Technology, 01 TL29, District 12, Ho Chi Minh City 700000, Vietnam; 3 NTT Hi-Tech Institute, Nguyen Tat Thanh University, Ho Chi Minh City 700000, Vietnam; blgiang@ntt.edu.vn (L.G.B.); ndchinh@ntt.edu.vn (D.C.N.); 4 Center of Excellence for Functional Polymers and NanoEngineering, Nguyen Tat Thanh University, Ho Chi Minh City 700000, Vietnam; 5 Institute of Applied Science, University of Technology, 475A Dien Bien Phu Street, Ward 25, Binh Thanh District, Ho Chi Minh City 700000, Vietnam; hongxuanlt289@gmail.com (T.H.X.L.); khanhhungpham92@gmail.com (K.H.P.); 6 Biomaterials and Nanotechnology Research Group, Faculty of Applied Sciences, Ton Duc Thang University, Ho Chi Minh City 700000, Vietnam

**Keywords:** antimicrobial activity, bacteriocin, food preservation, *Lactobacillus plantarum*, microencapsulation

## Abstract

Antimicrobial compounds from traditional fermented foods have shown activity against a wide range of pathogen and spoilage microorganisms for several years. In this study, a Lactic acid bacteria (LAB), isolated from Vietnamese traditional fermented yogurt (*Lactobacillus plantarum* SC01), was encapsulated in alginate-gelatin (ALG-GEL) and the effect of incubation temperature, medium pH and surfactants were assessed. The aims of this research were to evaluate antimicrobial activity of bacteriocin produced by *L. plantarum* SC01. Another aim the research was to study the quality of pork meat treated with its Bacteriocin in 2 h as a bio-preservative at different storage times (0 h, 12 h, 24 h and 48 h) in room temperature, compared to control (treated with salt 40.0%). The antimicrobial activity of *L. plantarum* SC01 was identified through the inhibition rate of five indicator organisms, including *Escherichia coli*, *Salmonella* sp., *Staphylococcus aureus*, *Listeria monocytogenes*, and *Bacillus subtilis* by co-culture method. The results showed that *L. plantarum* SC01 microencapsulated in ALG-GEL (2.5% alginate and 6.0% gelatin, *w*/*v*) and 3.0% bacteria supplied into modified MRS medium (MRSOPTSC01) produced highly active compound inhibited the growth of indicator organisms at a density of 10^4^–10^8^ CFU/mL. Antibacterial compounds were highly active in a treatment at 80 °C; not to be affected by pH; affected by surfactant as Ethylenediaminetetraacetic acid (EDTA), Sodium dodecyl sulfate (SDS), and Tween. Moreover, LAB obtained from this study show the potent Bacteriocin in its usage as a preservative in food.

## 1. Introduction

Chemical preservatives have been used for centuries to maintain freshness, improve visual properties, and prolong the shelf life of food. However, some previous studies reported that chemical additives in food cause a variety of adverse health effects, including cancer in humans [1,2,3,4,5,6]. Phosphoric acid, sodium nitrite, sodium benzoate, propyl gallate, aspartame, monosodium glutamate, butylated hydroxytoluene, potassium nitrite, ammonium sulfate, and titanium dioxide are among food preservatives that might be detrimental to human health [7]. Therefore, effective preservation techniques by other natural preservatives become an urgent need. The application of natural antimicrobials as food preservatives to prevent quality loss is a promising alternative.

Lactic acid bacteria (LAB) are known for the potential to produce antimicrobial compounds and are widely applied in the food industry, specifically in the manufacture of fermented foods. LAB are Gram-positive, non-spore forming, fermentative bacteria that grow anaerobically [8]. To date, some exopolysaccharides (EPSs), antimicrobial peptides (Bacteriocin), and Bacteriocin-like inhibitory substances (BLIS), produced by LAB, have shown potential health-benefits, such as an improved nutritional value, control of intestinal infection, and inhibition of pathogenic bacteria [9]. *Lactobacillus plantarum* is one of the most important LAB used for the production of fermented meat, grass, and vegetable products. Various Bacteriocins produced by *L. plantarum,* have been described, such as plantaricin A, plantaricin B, plantaricin C, plantaricin C19, plantaricin F, plantaricin S, plantaricin T, plantaricin LC74, plantaricin SA6, plantaricin 149, plantaricin 154, plantaricin UG1, and plantaricin KW30 [10].

Nowadays, new approaches for increasing bacterial cell concentration and improving their antimicrobial production are focused in recent research pathways, and microencapsulation techniques have been receiving special attention [11]. Microencapsulation is a method by which bacteria are protected from adverse environmental conditions, such as high acidity, bile salts, molecular oxygen in case of obligatory anaerobic microbes, bacteriophages, and chemicals, as well as antimicrobial agents [12,13,14]. The processing of a forming capsule was developed by techniques like co-accretion, co-crystallization, molecular inclusion, spray drying, spray cooling, chilling, extrusion, and fluidized bed drying. [11,15,16]. Currently, the choice of the technique depends on the type of the material to be microencapsulated, the application, and the release mechanism for its action.

Alginate and other gelling hydrocolloids are used as a material for cell encapsulation because they are non-toxic, biocompatible, and inexpensive [17,18]. Alginate is an anionic polymer obtained from brown seaweed, and is promising for cell protection. Alginate hydrogels can be prepared by addition of cations Ca^2+^, and at high calcium concentrations, multiple cross-linking among alginate chains is created [18,19,20]. Another important material for cell encapsulation is gelatin. Gelatin finds application in food, and pharmaceutical industry. Gelatin is derived from collagen, which has long been used in the food industry as a clarification agent, stabilizer, and protective coating material. Although various studies have shown that the entrapment of LAB in alginate hydrogels improves lactic acid production, little is known of BLIS production by microencapsulated LAB in alginate-gelatin (ALG-GEL) [21]. In this study, we investigated the effect of factors (temperature, pH, and surfactants) on antimicrobial activity of LAB isolated from Vietnamese traditional fermented yogurt in ALG-GEL capsules for higher inhibitory substances. Moreover, application in food preservation of Bacteriocin in situ or purified or semi-purified produced by *L. plantarum*, microencapsulated in ALG-GEL, may be an interesting technological aid to prolong the shelf life of products.

## 2. Materials and Methods

### 2.1. Bacterial Strains and Culture Conditions

LAB subjects were isolated from traditional fermented foods collected from a local market in Ho Chi Minh City (Vietnam), as described in a previous study. *L. plantarum* SC01 was isolated from Vietnamese traditional fermented yogurt following a standard serial dilution method and plating over selective Man Rogosa & Sharpe (MRS) agar. The identity of Gram-positive, catalase negative rods, i.e., tentative lactobacilli, were further examined by comparing the obtained sequences with those in the DNA database (http://www.ncbi.nim.nih.gov/blast). Five pathogenic strains used as the antimicrobial activity indicator, *Escherichia coli*, *Staphylococcus aureus*, *Bacillus subtilis*, *Salmonella* sp., and *Listeria monocytogenes* were supplied by the Research Institute for Aquaculture No. 2 (RIA2, Vietnam).

*L. plantarum* SC01 was maintained and propagated in MRSOPTSC01 broth (MRS medium optimized for Bacteriocin production). MRSOPTSC01 was supplemented with 10 g/L of beef extract, 5 g/L of yeast extract, 10 g/L of tryptone, 20 g/L of sucrose, 5 g/L of sodium acetate, 4 g/L of dipotassium phosphate, 2 g/L of ammonium citrate, 0.2 g/L of magnesium sulfate, 0.05 g/L of manganese sulfate, 1 mL/L of tween 80, and had its pH adjusted to 6.0. Antimicrobial activity indicator strains were grown at 37 °C for 24 h in Tryptone Soya Broth (TSB). All the AR grade chemicals used were purchased from HiMedia Laboratories Pvt. Ltd. (Mumbai, India); strains were stored at −80 °C in presence of 20% (*v*/*v*) glycerol.

### 2.2. Microencapsulation of L. plantarum SC01

*L. plantarum* SC01 was activated in MRSOPTSC01 broth at 37 °C for 48 h and propagated two times before being used in subsequent experiments. The culture was prepared by adding 1.0% inoculum to the MRSOPTSC01 broth and incubated at 37 °C for 24 h. The cells were collected by centrifugation at 4000 rpm at 4 °C for 15 min. After the removal of supernatants, the cells were microencapsulated by extruding a mixture containing *L. plantarum* SC01 suspended in sterilized wall materials (ALG-GEL solution, sterilized at 121 °C for 15 min) through a syringe and denatured into 0.1 M CaCl_2_ solution at room temperature (Distance between the syringe and CaCl_2_ solution was 10 cm). The beads were allowed to stand for 1 h for hardening, followed by rinsing with distilled water, filtering, and sealing in sterilized conical tubes for storage at 4 °C [22].

### 2.3. Bacteriocin Production

Bacteriocin produced from microencapsulated bacteria cells and free cells were cultivated in 250 mL MRSOPTSC01 at 37 °C for 48 h. Bacteriocin with free cells was used as a control.

### 2.4. Antimicrobial Activity of Bacteriocin-Containing Supernatants

The antimicrobial activity was evaluated by co-culture method [23]. Briefly, 1.0% cells were microencapsulated in ALG-GEL were added into MRSOPTSC01 broth and incubated at 37 °C for 48 h. The cell-free supernatant (CFS) was recovered and sterilized by filtration through Millex-GV 0.22 μm hydrophilic Durapore PVDF membrane (Millipore, Billerica, MA, USA) and then, adjusted to pH 6.0 with 2 N NaOH in order to rule out inhibitory effects due to organic acids. The neutralized CFS were mixed 1:1 with TSB medium, and bioindicator overnight cultures (0.05%, *v*/*v*). Co-culture incubated for 24 h at 37 °C, and the growth of bioindicator was monitored by measuring optical density at 600 nm (OD_600_).

### 2.5. Effect of Different Factors on Bacterial Growth

Initially, the cultivation of *L. plantarum* SC01 for Bacteriocin production was conducted in aerobic conditions. Then the cultivation was carried out in 250 mL flasks containing 100 mL of MRSOPTSC01 medium. The flasks were inoculated with 1.0% inoculum and incubated in an anaerobic jar at 37 °C for 24 h. After centrifugation, the cells were microencapsulated by extruding a mixture containing *L. plantarum* SC01 suspended in sterilized wall materials. Several ALG-GEL solutions including 2.5% ALG-6.0% GEL, 2.5% ALG-8.0% GEL, 2.5% ALG-10.0% GEL, 2.5% ALG-12.0% GEL and 2.5% ALG-14.0% GEL were assessed to identify the preferred wall material for the production of Bacteriocin [17,19]. In each case, 2.5% alginate was used as a primary wall material source while gelatin was used as additional wall material.

The effect of initial culture inoculum concentrations on Bacteriocin production by microencapsulated *L. plantarum* SC01 in ALG-GEL was evaluated at 37 °C for 48 h using MRSOPTSC01. The initial culture inoculum concentrations of *L. plantarum* SC01 were ranging from 1% to 4% [14].

The effect of bioindicator concentrations on Bacteriocin production by *L. plantarum* SC01 were also studied in gram-negative and gram-positive bacteria. For bioindicator cultivation, antimicrobial activity indicator strains were grown at 37 °C for 24 h in TSB with concentrations (10^4^, 10^5^, 10^6^, 10^7^, and 10^8^ CFU/mL) [21]. Percentage of inhibition was expressed as inhibition (%) of bioindicator growth relative to the control (i.e., bioindicator grown in optimal conditions, without CFS) [23]:

(1) $$\%\;\mathrm{inhibition}=\left(\frac{cfu/ml\;in\;control\;-\;cfu/ml\;in\;co-incubation\;culture}{cfu/ml\;in\;control}\right)\times \;100\%$$

### 2.6. Stability of the Antimicrobial Activity at Different Temperature, pH, and Surfactants

The neutralized CFS were treated at 37 °C, 60 °C, 80 °C, and 100 °C for 30 min, and then assayed for residual activity. The sensitivity of the antagonistic compound to different pH was estimated by adjusting the pH of supernatant to varying pH values, ranging between 2.0 and 10.0 with 2 N NaOH or 2 N HCl. Following that, the supernatant was incubated at 37 °C for 2 h and was evaluated for residual activity. The effect of surfactants was evaluated after concentration and treatment of active supernatants with 1.0% (*w*/*v*) SDS, Tween 20, Tween 80, Urea, and EDTA. Controls consisted of either active supernatant or detergents used and samples were incubated at 37 °C for 5 h and then tested for activity.

### 2.7. Bacteriocin Application for Pork Meat Preservation

Fresh pork meat samples were taken for the evaluation and split into two independent experiments. The experimental samples were treated with Bacteriocin-containing supernatants and the other experimental samples were treated with salt 40.0%, which serves as a control. The experimental and control pork samples were stored at an ambient temperature, and then tested by sensory analysis (nine consumer panelists of University of Technology consisting of 6 females and 3 males of 22–35 years of age were recruited). Sensory evaluation pork meat samples treated with Bacteriocin-containing supernatants for 2 days was subjected to consumer acceptability testing. Panelists evaluated appearance, odor, texture, taste, and overall acceptability of meat using a 9 point hedonic scale of 1 = extremely dislike, 2 = dislike very much, 3 = dislike, 4 = slightly dislike, 5 = neither like nor dislike, 6 = a bit like, 7 = like, 8 = like very much, 9 = extremely like [24].

In addition, in vitro tests of the probiotic activity were performed as follows. Pork meat samples, treated with Bacteriocin-containing supernatants, and the control were examined on a cultured pathogenic bacteria (Total Bacterial Count (TBC), Coliform bacteria, *S. aureus*, *E. coli*, and *Salmonella*) at 0 h, 12 h, 24 h, and 48 h to assess its effect on the pathogen.

### 2.8. Statistical Analysis

Three independent experiments were conducted for all experiments and results were reported in mean value ± standard deviation (SD). All statistical study was performed using Statgraphics Centurion software program (Statpoint Technologies, Inc., Warrenton, VA, USA). The Student’s *t* test was used to compare the significance of differences that were considered significant at the level of *p* < 0.05.

## 3. Results

### 3.1. Effects of Different Gelatin Concentrations, Bacterial Inoculation Rates and Bioindicator Concentrations on Bacteriocin Production

According to a previous study, low concentrations (less than 2%, *w*/*v*) of alginate used as the supporting gel or their mixture with other gels resulted in low viscosity and crosslinking sites did not create an uniform microencapsulated capsule. Whereas, the extrusion of alginate, or their mixture with other gels through a syringe at the high concentrations (3%, *w*/*v*), was difficult due to high viscosity [3]. Hence, the present study selected 2.5% (*w*/*v*) sodium alginate as the optimum concentration of biopolymer matrices ALG-GEL. *L. plantarum* SC01 was microencapsulated in ALG-GEL and tested for its antimicrobial activity. The effects of different gelatin concentrations (6%, 8%, 10%, 12%, and 14% *w*/*v*), different bacterial inoculation rates (1%, 2%, 3%, and 4% *w*/*v*), and different bioindicator concentrations (10^4^, 10^5^, 10^6^, 10^7^, and 10^8^ CFU/mL) on Bacteriocin production of microencapsulated LAB were investigated. The effect of gelatin concentration mixed with 2.5% alginate on antimicrobial activity of Bacteriocin-containing supernatants are shown in Table 1. In particular conditions, the formation of a mixed gel of alginate and gelatin is obtained.

There was a significant increase (*p* < 0.05) in bio-indicator growth, compared to different gelatin concentrations under similar conditions. An increase in gelatin concentration mix, during microencapsulation, increased the growth of bio-indicators. This study clearly showed that *L. plantarum* SC01 was microencapsulated in ALG-GEL (2.5% (*w*/*v*) alginate and 6.0% (*w*/*v*) gelatin), producing highly active compound that inhibited the growth of bio-indicator. The increasing of gelatin concentration will increase the viscosity of the solution and cause difficulties in the process of wrapping, thus greatly influences the quality of the package. This also greatly affects the fertility of antimicrobial compounds of bacteria [25]. Al-muhanna et al. (2015) reported that, as the concentration of gelatin increases, its solubility in the liquid gelatin-alginate will be reduced, worsening the ability to link with alginate gel [26]. Meanwhile, another study showed that the gelation time of ALG-GEL decreased with increasing gelatin content and buffer solution with Ca^2+^ had a significant effect on the release of ALG-GEL capsules [27].

Regarding the bacteria inoculation rate, it should be noted that the antagonistic compound produced by LAB was due to the bacterial inoculation rate [28]. For instance, Silva et al. showed that three Bacteriocin-producing strains had high antimicrobial activity when inoculating the LAB at 1.0% (*v*/*v*) into MRS broth medium [29]. In this study, four bacterial concentrations (1%, 2%, 3%, and 4%, *v*/*v*) were conducted in an attempt to identify a novel approach to optimize bacterial inoculation rate in producing antibacterial compounds. *L. plantarum* SC01 was grown in MRSOPTSC01 broth at 37 °C for 48 h. CFS were microencapsulated in ALG-GEL (2.5% alginate and 6.0% gelatin, *w*/*v*), and the effect of *L. plantarum* SC01 inoculation rate on Bacteriocin production are showed in Table 2.

The highest percentages of inhibition were recorded at *L. plantarum* SC01 concentrations at 3% and 4% (*v*/*v*). To be specific, mean resistance to *E. coli*, *S. aureus*, *B. subtilis* respectively peaked at 56.85%, 56.79%, and 61.31%, corresponding to 3% *L. plantarum* SC01 inoculated. Against *Salmonella*, and *L. monocytogenes*, compared to other rates, 4% *L. plantarum* SC01 seems to inhibit the two bacteria the most, at mean rates of 63.27%, and 55.38%. However, except for results for *S. aureus*, differences between inhibitions produced by *L. plantarum* SC01 inoculated at the rate of 3% and 4% were not statistically significant, suggesting that increasing inoculation rate from 3% to 4% yielded diminished inhibitory effects. In an attempt to facilitate large-scale industrial production, inoculation rate of 3.0% was chosen to test the effect of factors on LAB antimicrobial activity.

Table 3 shows the growth of *E. coli*, *Salmonella*, *S. aureus*, *B. subtilis*, and *L. monocytogenes* at an initial level of 10^4^–10^8^ CFU/mL inhibited by Bacteriocin produced by *L. plantarum* SC01 microencapsulated in ALG-GEL.

For *Salmonella* and *S. aureus* (10^4^ CFU/mL), it was shown that Bacteriocin allowed initial growth of the bio-indicators, with the low and medium concentration (10^4^–10^7^ CFU/mL), and inhibited the growth of the pathogens with the highest concentration of 10^8^ CFU/mL. For the other bacteria, the inhibitor percentages are not significantly different at the initial level of 10^4^–10^8^ CFU/mL. This experiment showed that the inhibitory ability of antimicrobial compounds, produced by *L. plantarum* SC01, was independent of the concentration of bio-indicator organisms. Meanwhile, previous studies have shown that the percentage of inhibition decreased when the concentration of indicator organisms increased [30]. In this study, antimicrobial compounds produced by *L. plantarum* SC01 is effective in inhibit pathogenic bacteria growth, whereas bio-indicator concentrations can be as dense as 10^8^ CFU/mL.

### 3.2. Effect of Temperature, pH and Surfactants on Antimicrobial Activity

Different temperatures (37 °C, 60 °C, 80 °C, and 100 °C) were chosen to treat the CFS for 30 min and the percentage of antimicrobial activity are shown in Table 4. The maximum percentage of inhibition reached approximately 60.0% after Bacteriocin-containing supernatants were treated at 80 °C and reached about 50.0% after being treated at 60 °C for 30 min. On the contrary, thermal treatment at 100 °C resulted in the loss of antimicrobial activity.

The result suggests that Bacteriocin-containing supernatants should be treated at temperature of 80 °C for the purpose of pathogen elimination and/or bacteria and bacterial spore removal. Similar results were recorded for a number of Bacteriocin, produced by *Lactobacillus* spp. and lactocin NK24 produced by *Lc. lactis* NK24, where the loss of its activity after 30 min at 100 °C achieved 87.5% [31,32]. In another study where lactocin MMFII was produced by *Lc. lactis* MMFII, only 8.3% activity was recorded after 30 min at 110 °C and 25% after 30 min at 80 °C and 90 °C [33]. Nisin, which is produced by *Lc. lactis* subsp. *lactis* WNC20, was incubated at 121 °C, pH 7.0 and then inactivated after 15 min whereas the sample that is incubated at pH 3.0 was not inactivated [34]. Bozacin B14, produced by *Lc. lactis* subsp. *lactis* B14, was also inactivated after 10 min at 90 °C [35].

Initial pH medium exerted a minor influence on production of *L. plantarum* SC01 (Table 5). Optimal levels of Bacteriocin were produced in MRSOPTSC01 at pH of 4.0. The percentage of antimicrobial activity reached 60.22% for *E. coli* and reached around 50.0% for other bio-indicator. However, with the increase of pH from 2 to 10, the percentage of inhibition exhibited the same behavior. A similar study, Bacteriocins ST22Ch, ST153Ch and ST154CH, produced by three strains of *Lactobacillus sakei*, isolated from salpicao, a fermented meat product from North-West of Portugal have a narrow spectrum of activity, are heat resistant and stable between pH 2.0 and pH 10.0 [32].

Different surfactants were tested including Sodium dodecyl sulfate (SDS), Tween 20, Tween 80, Urea, and EDTA at final concentration of 1.0% (*w*/*v*) [36,37]. The highest percentage of inhibition was observed at EDTA while the lowest percentage of inhibition was observed at urea (Table 6). In previous studies, Todorov et al. showed that activity of Bacteriocins was not affected by treatment with 1% Triton X-100, Tween 20, Tween 80, SDS, NaCl, urea and EDTA [32]. The present study indicated that Bacteriocin could improve antimicrobial activity when treated with 1.0% (*w*/*v*) EDTA.

Several studies have demonstrated the diverse effects of LAB-loaded microencapsules, based on alginate gelatin, validating its potential use as a functional food. Microencapsulation of LAB and its antimicrobial potentiality had been observed by Li et al., Khandrae et al., Ariza et al., Corbo et al., Mei et al., and Léonard et al. [17,18,38,39,40,41]. Li et al. reported that encapsulation in alginate gelatin microcapsules successfully improved the survival of *L. casei* ATCC 393 and could increase the cell numbers to 10^7^ CFU/g in the dry state of microcapsules [40]. In this way, Bacteriocin produced by *Lactobacillus plantarum* microencapsulated in ALG-GEL capsules showed the potential as antagonistic compound.

### 3.3. Effect of Bacteriocin on Pork Meat Preservation

**Figure 1:**
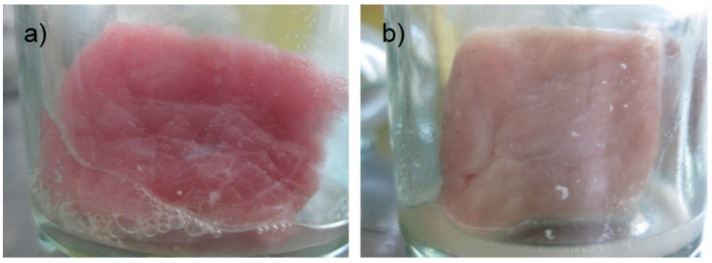
(**a**) Control sample and (**b**) sample after 24 h.

According to physical quality of pork meat during storage, after 12 h, the control sample started to emit odor and change in color, while the experiment samples treated with Bacteriocin-containing supernatants were odorless, elastic and had fresh, clean, and dry surfaces. Control samples at 24 h and 48 h could not maintain its original state, as well as impurities (Figure 1). Meanwhile, experimental samples began to show signs of decomposition after 48 hours. This may be due to experimental meat samples are soaked in service only a single antibiotic should extend the survey period, the antibacterial activity also lost thereby not limiting the growth of bacteria present in the sample.

**Figure 2:**
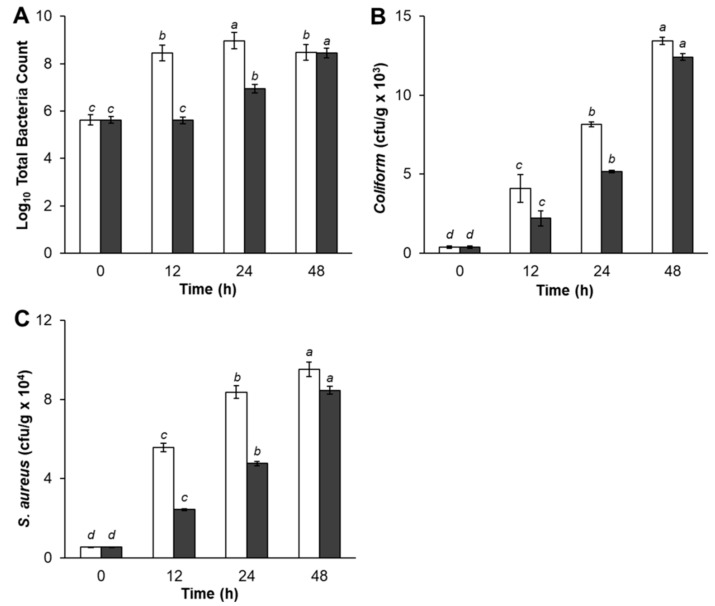
(**A**) Total Bacterial Count (TBC), (**B**) Coliform bacteria and (**C**) *S. aureus*. Results are the mean of two independent experiments. Vertical bars indicate standard error; white bars, control; black bars, sample (^a, b, c, d^ means with the same letter in the same row are not significantly different at *p* < 0.05).

The microbial analyses of treated pork meat samples are shown in Figure 2. As shown in Figure 2A, TBC of control pork meat was significantly higher after storage for 12 h and 24 h, compared with experimental samples treated with Bacteriocin-containing supernatants. However, experimental samples TBC gradually increased by 24 h of storage period, then dramatically increased at the end of storage time. There was no significant difference between control and pork meat treatments in TBC at 48 h. Pork meat samples treated with Bacteriocin produced by *L. plantarum* SC01 exhibited a slight increase in TBC (2.9 × 10^8^, CFU/g) at 12 h compared with control (4.0 × 10^5^, CFU/g). The inhibition of pathogenic bacteria growth was decreased after 24 h, and after 48 h, had the lowest rate of antimicrobial activity which TBC in control sample was 2.9 × 10^8^ (CFU/g), while the samples were 2.7 × 10^8^ (CFU/g), respectively. The same result was in agreement with Hamid et al. (2007), who reported that, during the storage of pork in nisin, TBC slightly increased during the first period of storage and then significantly increase till the end of storage period. That increase could be evidentially referred to the decrease in Bacteriocin production, which controls the rate of growth. This result indicated that adding Bacteriocin in pork meat had an inhibitory effect on pathogenic bacteria and the inhibition was apparent in fresh pork until it reached a maximum inhibition at 12 h of storage period.

Regarding the result of Total Coliform, data in Figure 2B showed that Coliform bacteria was presented in both control and samples treated with Bacteriocin production. The control sample (4.1 × 10^3^, CFU/g) had a significantly higher number of Coliform bacteria count at 12 h storage period as compared with the sample treatment (2.2 × 10^3^, CFU/g). The Total Coliform increased after 24 h and 48 h. The quantity of Coliform bacteria in the samples increased, but lower than the control sample, indicating that antagonistic compound produced by *L. plantarum* SC01 limited the growth of Coliform bacteria in samples. Our result was in accordance with Kim et al. (2004), who stated that fresh pork loins sprayed with organic acids, retarded pathogenic bacteria growth [42]. This is obviously due to recontamination and suppressive effect of added starter cultures and probiotic *L. plantarum* SC01 on Coliform bacteria.

Figure 2C showed that Bacteriocin-containing supernatants seem to prevent the growth of *S. aureus*. During storage period, *S. aureus* in treated sample gradually decreased after 12 h of storage period and insignificantly decreased at the end of the storage period compared with the control, in which the *S. aureus* counts increased rapidly. At 12 h, *S. aureus* in control sample was 5.6 × 10^4^ (CFU/g). This figure in the treated sample was 2.4 × 10^4^ (CFU/g) and increased after 24 h and 48 h. This result also showed that S. *aureus* in samples were under control when treated with Bacteriocin. As predicted, antagonistic compound produced by *L. plantarum* SC01 limited the growth of *S. aureus*. Hereupon, the inhibitory effect upon *S. aureus* organisms in treatment pork meat could be attributed to the attained high acidity of acidic metabolites end products, such as (lactic-acetic acids) and the Bacteriocin produced by *L. plantarum* SC01. The results in this study indicated that samples were negative for *E*. *coli* and *Salmonella*. At the same point of view, Dabiza et al. added the CFS of *L. rhamnosus* showed the highest effect among *Lactobacilli* against *E. coli*, *S. aureus*, *B. cereus*, and *Aeromonas hydrophila* [43].

The result showing that antagonistic compound, produced by *L. plantarum* SC01, exhibited the highest capacity to preserve pork meat at 12 h. Because at this time, the antimicrobial compound remained active, inhibited the development of bacteria in the sample and limited the density of bacteria. By the time 24 h and 48 h, the bacteria began growth strongly, while the efficiency of antimicrobial compound diminished, creating favorable conditions for bacteria to develop.

The results from this study indicated that these antimicrobial compounds are only suitable for short-term storage. To be specific, the preservation time of pork meat should not exceed 2 days when stored in room temperature, which limits its potential application as bio-preservative. However, the results of this study will be a prerequisite for future developments of biological preservatives to improve the safety for users and increase usage time of food products.

## 4. Conclusions

The microencapsulation of LAB has proven to be an alternative for the induction of Bacteriocin production. In the case of *L. plantarum* SC01, isolated from Vietnamese traditional fermented yogurt, it has been shown that *L. plantarum* SC01 microencapsulated in ALG-GEL (2.5–6.0%, *w*/*v*), and supplied with 3.0% bacteria into the MRSOPTSC01 medium. This generated an increase in the percentage of antimicrobial activity, as evidenced in the Bacteriocin, which was highly active when treated at 80 °C, using EDTA surfactant, and were not affected by pH. ALG-GEL formulation also inhibited the growth of pathogenic bacteria in pork meat throughout the 12 h period. Thus, our results indicate that *L. plantarum* SC01 from Vietnamese traditional fermented yogurt might be a suitable candidate probiotic for promoting food preservation.

## Figures and Tables

**Table 1:** Effect of different gelatin concentrations on percentage of inhibition (%).

	Alginate-Gelatin 6.0%	Alginate-Gelatin 8.0%	Alginate-Gelatin 10.0%	Alginate-Gelatin 12.0%	Alginate-Gelatin 14.0%
*E. coli*	70.17 ± 2.52 ^a^	44.44 ± 3.14 ^b^	38.56 ± 1.82 ^c^	16.47 ± 2.82 ^d^	14.81± 7.41 ^d^
*Salmonella*	63.39 ± 4.44 ^a^	40.13 ± 1.10 ^b^	34.26 ± 2.73 ^c^	13.09 ± 2.98 ^d^	5.53 ± 4.86 ^e^
*S. aureus*	64.50 ± 2.49 ^a^	41.56 ± 0.99 ^b^	33.33 ± 2.70 ^c^	11.97 ± 0.86 ^d^	7.25 ± 4.09 ^e^
*B. subtilis*	69.50 ± 1.65 ^a^	42.64 ±3.11 ^b^	36.50 ± 1.13 ^c^	15.47 ± 5.27 ^d^	13.33 ± 2.63 ^d^
*L. monocytogenes*	68.69 ± 3.01 ^a^	44.84 ± 1.26 ^b^	38.32 ± 2.12 ^b^	19.13 ± 4.06 ^c^	18.83 ± 3.23 ^c^

^a, b, c, d^ means with the same letter in the same row are not significantly different at *p* < 0.05.

**Table 2:** Effect of different *L. plantarum* SC01 inoculation rates on percentage of inhibition (%).

	1.0%	2.0%	3.0%	4.0%
*E. coli*	8.91 ± 1.01 ^c^	38.25 ± 0.70 ^b^	56.85 ± 0.59 ^a^	55.32 ± 1.11 ^a^
*Salmonella*	21.47 ± 0.83 ^c^	42.19 ± 1.53 ^b^	61.45 ± 0.79 ^a^	63.27 ± 0.87 ^a^
*S. aureus*	6.56 ± 0.62 ^d^	36.66 ± 0.96 ^c^	56.79 ± 1.33 ^a^	53.28 ± 3.14 ^b^
*B. subtilis*	18.21 ± 3.16 ^c^	42.80 ± 0.94 ^b^	61.31 ± 1.52 ^a^	60.22 ± 0.92^a^
*L. monocytogenes*	15.60 ± 1.49 ^c^	32.12 ± 1.07 ^b^	54.70 ± 0.99 ^a^	55.38 ± 0.59^a^

^a, b, c, d^ means with the same letter in the same row are not significantly different at *p* < 0.05.

**Table 3:** Effect of different bioindicator concentrations on percentage of inhibition (%).

	10^4^ CFU/mL	10^5^ CFU/mL	10^6^ CFU/mL	10^7^ CFU/mL	10^8^ CFU/mL
*E. coli*	64.81 ± 2.56 ^ab^	65.87 ± 1.65 ^a^	63.51 ± 0.47 ^b^	61.41 ± 1.25 ^c^	66.32 ± 0.92 ^a^
*Salmonella*	44.73 ± 3.51 ^c^	48.97 ± 0.15 ^b^	48.57 ± 1.08 ^b^	49.47 ± 2.82 ^b^	57.02 ± 1.39 ^a^
*S. aureus*	38.84 ± 1.67 ^b^	39.60 ± 3.02 ^b^	43.36 ± 2.55 ^a^	34.94 ± 3.37 ^c^	46.02 ± 2.22 ^a^
*B. subtilis*	49.89 ± 2.11 ^b^	51.63 ± 7.08 ^ab^	52.76 ± 1.48 ^a^	49.94 ± 1.80 ^b^	53.49 ± 5.14 ^a^
*L. monocytogenes*	46.82 ± 1.36 ^b^	48.13 ± 1.59 ^b^	49.11 ± 3.24 ^ab^	47.07 ± 2.23 ^b^	51.00 ± 1.77 ^a^

^a, b, c, d^ means with the same letter in the same row are not significantly different at *p* < 0.05.

**Table 4:** Effect of temperature on antimicrobial activity (%).

	30 °C	60 °C	80 °C	100 °C
*E. coli*	43.49 ± 2.19 ^c^	52.41 ± 1.22 ^b^	55.31 ± 1.92 ^b^	19.91 ± 1.45 ^d^
*Salmonella*	42.29 ± 1.45 ^c^	47.32 ± 0.77 ^b^	60.40 ± 1.86 ^a^	17.35 ± 0.74 ^d^
*S. aureus*	41.86 ± 1.02 ^c^	51.82 ± 0.57 ^b^	57.98 ± 4.58 ^a^	12.37 ± 0.53 ^d^
*B. subtilis*	40.39 ± 0.97 ^c^	43.56 ± 1.78 ^c^	55.96 ± 0.51 ^a^	11.48 ± 0.98 ^d^
*L. monocytogenes*	40.50 ± 1.47 ^c^	51.54 ± 1.69 ^b^	58.91 ± 1.09 ^a^	18.07 ± 1.29 ^d^

^a, b, c, d^ means with the same letter in the same row are not significantly different at *p* < 0.05.

**Table 5:** Effect of pH on antimicrobial activity (%).

	*E. coli*	*Salmonella*	*S. aureus*	*B. subtilis*	*L. monocytogenes*
pH 2	51.42 ± 1.69 ^bc^	49.66 ± 1.90 ^ab^	49.67 ± 0.68 ^cd^	50.67 ± 1.89 ^a^	47.97 ± 1.52 ^bc^
pH 3	52.31 ± 1.06 ^b^	51.33 ± 0.73 ^ab^	52.36 ± 1.83 ^b^	51.73 ± 2.00 ^a^	50.26 ± 1.71 ^ab^
pH 4	60.22 ± 1.24 ^a^	52.84 ± 0.71 ^a^	59.80 ± 0.50 ^a^	51.51 ± 1.84 ^a^	50.49 ± 2.08 ^a^
pH 5	51.27 ± 0.94 ^bc^	47.37 ± 1.75 ^b^	50.17 ± 1.81 ^bcd^	46.67 ± 0.69 ^b^	47.11 ± 1.38 ^c^
pH 6	51.56 ± 1.13 ^bc^	53.64 ± 2.00 ^a^	51.47 ± 1.51 ^bc^	50.87 ± 0.84 ^a^	51.23 ± 0.45 ^a^
pH 7	50.30 ± 1.89 ^bcd^	39.65 ± 0.79 ^de^	50.43 ± 1.54 ^bcd^	43.52 ± 0.95 ^c^	45.82 ± 1.48 ^cd^
pH 8	48.17 ± 0.94 ^d^	41.33 ± 1.17 ^d^	50.26 ± 0.56 ^bcd^	50.59 ± 1.77 ^a^	46.99 ± 0.33 ^c^
pH 9	49.43 ± 1.89 ^cd^	37.78 ± 0.45 ^e^	49.87 ± 1.58 ^cd^	45.45 ± 1.85 ^bc^	44.57 ± 1.07 ^d^
pH 10	50.11 ± 1.25 ^bcd^	44.05 ± 1.80 ^c^	48.74 ± 1.46 ^d^	49.66 ± 1.62 ^a^	45.98 ± 1.66 ^cd^

^a, b, c, d^ means with the same letter in the same row are not significantly different at *p* < 0.05.

**Table 6:** Effect of 1.0% (*w*/*v*) of surfactants on antimicrobial activity (%).

	Control	Tween 80	Tween 20	EDTA	Urea	SDS
*E. coli*	83.66 ± 1.60 ^b^	10.03 ± 1.81 ^e^	25.66 ± 0.82 ^d^	95.47 ± 0.48 ^a^	0.00	65.78 ± 1.24 ^c^
*Salmonella*	72.32 ± 0.54 ^b^	14.17 ± 0.37 ^e^	36.00 ± 1.96 ^d^	98.42 ± 0.68 ^a^	0.00	60.21 ± 2.21 ^c^
*S. aureus*	72.93 ± 1.82 ^b^	10.19 ± 0.85 ^e^	21.79 ± 1.18 ^d^	92.32 ± 0.23 ^a^	0.00	37.20 ± 1.96 ^c^
*B. subtilis*	70.86 ± 2.04 ^b^	23.06 ± 1.99 ^d^	35.23 ± 2.21 ^c^	98.47 ± 0.58 ^a^	0.00	14.64 ± 1.04 ^e^
*L. monocytogenes*	62.96 ± 1.82 ^b^	11.39 ± 0.82 ^e^	25.92 ± 2.03 ^d^	93.87 ± 1.77 ^a^	0.00	51.71 ± 2.08 ^c^

^a, b, c, d^ means with the same letter in the same row are not significantly different at *p* < 0.05.
